# Specificity of the bilingual advantage for memory: examining cued recall, generalization, and working memory in monolingual, bilingual, and trilingual toddlers

**DOI:** 10.3389/fpsyg.2014.01369

**Published:** 2014-12-02

**Authors:** Natalie H. Brito, Amanda Grenell, Rachel Barr

**Affiliations:** ^1^Robert Wood Johnson Foundation Health and Society Scholars, Columbia University in the City of New YorkNew York, NY, USA; ^2^Department of Psychology, Georgetown UniversityWashington, DC, USA

**Keywords:** memory, bilingualism, infant development, deferred imitation, imitation, generalization, memory flexibility

## Abstract

The specificity of the bilingual advantage in memory was examined by testing groups of monolingual, bilingual, and trilingual 24-month-olds on tasks tapping cued recall, memory generalization and working memory. For the cued recall and memory generalization conditions, there was a 24-h delay between time of encoding and time of retrieval. In addition to the memory tasks, parent-toddler dyads completed a picture-book reading task, in order to observe emotional responsiveness, and a parental report of productive vocabulary. Results indicated no difference between language groups on cued recall, working memory, emotional responsiveness, or productive vocabulary, but a significant difference was found in the memory generalization condition with only the bilingual group outperforming the baseline control group. These results replicate and extend results from past studies (Brito and Barr, 2012, 2014; Brito et al., 2014) and suggest a bilingual advantage specific to memory generalization.

## Introduction

In many parts of the world, bilingualism or multilingualism is the norm and being monolingual is rare (Dutcher and Tucker, 1994; Grin, 2004). Within the context of research, however, bilingual participants are often treated as a special population with unique advantages or disadvantages from monolinguals. Not only is multilingualism a common occurrence, the ease with which children can acquire multiple languages (Bialystok, 1991; Kuhl, 2004; Paradis et al., 2010) indicates that humans are adept at processing this type of linguistic input. The influence of multiple languages on cognitive development has received increased attention in the last 15 years, but the majority of research has focused on executive functioning and its correlated constructs of inhibition, task switching, and attentional control (Miller and Cohen, 2001).

Researchers have argued that because bilinguals have two “active” languages they must inhibit one language when producing the other, thereby practicing attentional control at an earlier age (Green, 1998; Bialystok, 1999). Support for this model is provided by extensive research demonstrating specific bilingual advantages within the executive function system (Bialystok, 1999; Bialystok and Martin, 2004; Bialystok et al., 2005; Carlson and Meltzoff, 2008; Poulin-Dubois et al., 2011). Refining this control during the early years of a bilingual child's development is necessary for successful bilingual language acquisition. If this model is accurate, then bilingual children experience extensive practice of these functions from early in development, but this practice may not only come from the production of two languages but also from the exposure to them. For example, Kovács and Mehler (2009) used eye-trackers within an anticipatory cue cognitive control paradigm and found that bilingual 7-month-old infants were better than monolingual infants of the same age at using a novel cue to switch their attention to the correct location. These results suggest that simply perceiving and processing sounds from multiple native languages early in life leads to a domain-general enhancement of executive functions.

A literature search on cognitive development studies conducted between 2000 and 2013 with typically developing dual language learners (ages 0–6) generated approximately 100 peer-reviewed articles (Barac et al., 2014); 75% of those studies examined executive function or metalinguistic abilities and only a few studies have set out to specifically investigate memory abilities in bilingual children (Lanfranchi and Swanson, 2005; Messer et al., 2010) or bilingual infants (Brito and Barr, 2012). Recent studies have further supported a link between bilingualism and enhanced non-linguistic memory generalization abilities at 6- and 18-months of age (Brito and Barr, 2014; Brito et al., 2014). In the original study, Brito and Barr (2012) used the well-established deferred imitation puppet task to test 18-month-olds from various language backgrounds on a memory generalization task. In this paradigm, the experimenter demonstrates three target actions with one puppet (e.g., duck) to the infant, then after a 30-min delay, tests the infant with a novel puppet (e.g., cow). Results indicated that 18-month-old bilinguals, but not monolinguals, were more likely to generalize across puppets and recall the previously demonstrated target actions. In a subsequent study, these results were replicated with groups of infants exposed to two typologically similar (Spanish-Catalan) and two typologically different (English-Spanish) languages. Both bilingual groups of infants outperformed the monolingual groups, and there was no difference in memory generalization performance between the bilingual groups. Interestingly, infants exposed to three languages from birth (trilinguals) did not demonstrate an advantage in memory generalization, as their performance was no different from the baseline control or monolingual groups (Brito et al., 2014). In the present study, we examine the specificity of the bilingual advantage in memory by testing groups of monolingual, bilingual, and trilingual toddlers on tasks tapping cued recall, memory generalization, and working memory.

### Cued recall

To assess memory during infancy and toddlerhood, non-verbal measures are necessary. Deferred imitation tasks have been used in many past studies as a tool to examine cued recall in young infants. This paradigm capitalizes on an infant's propensity to imitate and studies have demonstrated that infants learn and recall novel action sequences demonstrated by an adult (e.g., Meltzoff, 1985; Barr et al., 1996), a peer (Hanna and Meltzoff, 1993), or even a televised model (Barr and Hayne, 1999). In this task an experimenter models a series of actions during the demonstration phase and the infant is not given an opportunity to interact with the objects or provided with verbal cues at any time. Additionally, the length of delay between demonstration and test is manipulated to increase or decrease cognitive load. During the test phase, the infants are given the stimuli from the previous demonstration and encouraged to play with them and infants are assessed on the number of target actions they can recall. Performance is compared to infants in the baseline control group who are not shown the demonstration, but simply given the stimuli during the test phase as their performance is used as an index of spontaneous production of the target behaviors. Deferred imitation is operationally defined as the experimental group performance significantly exceeding that of the baseline control group.

Traditional Piagetian theories on the emergence of deferred imitation were challenged when studies demonstrated that infants younger than 18-months (9- and 14-month-olds) were capable of deferred imitation after a 24-h delay (Meltzoff, 1985, 1988). Barr et al. (1996) demonstrated that 12−, 18−, and 24-month-olds were able to recall target actions after a 24-h delay, but there was no evidence of deferred imitation by 6-month-olds. When task parameters were altered, employing immediate imitation or increasing the duration of the demonstration phase, even 6-month-olds were capable of recalling previously seen target actions (Barr et al., 1996). Meltzoff (1985) found that infants as young as 14-months of age were able to recall a sequence of events after a lengthy 4-month delay, although there was evidence of a decline in the number of target actions remembered, suggesting some forgetting over time.

Researchers generally assume that a memory is a hypothetical collection of attributes that represent what the subject noticed at the time of original encoding (Estes, 1973, 1976; Spear, 1978; Roediger, 2000) and the *encoding specificity principle* assumes that the memory of the target event will be retrieved only if the cues encountered at retrieval match the same attributes seen during the original representation (Tulving and Thomson, 1973; Tulving, 1983, 1984). This has been supported by many studies demonstrating that changes in either stimuli or environmental context at the time of retrieval significantly disrupt memory performance (Godden and Baddeley, 1975; Tulving, 1983). That is, in order for an object to cue retrieval, the infant must recognize the similarity between the test object and the attributes stored as part of the original memory representation. Early in development the match between the encoding object and the test object must be nearly veridical, resulting in memory specificity being a robust feature of early memory processing. This may be adaptive because infants have very poor levels of inhibitory control (Diamond, 1990) and memory specificity therefore is a protective mechanism to keep infants from potential harm caused by responding to stimuli that may differ from those that they have originally encountered (Rovee-Collier, 1996). It may be as important for young children to demonstrate memory specificity in appropriate learning situations as it is for them to become more cognitively flexible across time (Bahrick, 2001; Learmonth et al., 2004). Failure, however, to develop memory flexibility across time will also become a maladaptive strategy and at its extreme may be exhibited in delayed cognitive development (Bauer, 2007; Riggins et al., 2009).

A few prior studies have examined short-term memory in the context of language abilities for young bilingual children. Thorn and Gathercole (1999) assessed phonological short-term memory in 5-year-old children and results indicated that performance for both monolinguals and bilinguals was dependent on vocabulary knowledge in their native languages. Lanfranchi and Swanson (2005) examined Spanish-English bilingual 6-year-olds and found that both phonological short-term memory (Digit Span) and working memory (immediate verbal free recall) were both language dependent for dual language learners, supporting Thorn and Gathercole's (1999) results; bilingual performance was not compared to monolingual performance in this study. Messer et al. (2010) found no differences between monolingual and bilingual 4-year-olds in their short-term memory task, and consistent with previous studies, language abilities did predict performance for both groups. No studies to our knowledge have examined non-verbal cued recall for multilingual infants.

### Memory generalization

A hallmark of memory development during the infancy period is an age-related increase in the flexibility of memory retrieval. Memory may start off highly specific, but memory flexibility or generalization gradually improves as the infant develops (Hayne, 2006; Barr and Brito, 2014). For example, although 12-month-olds who are tested in the deferred imitation puppet task imitate the target actions when tested in a novel context (Hayne et al., 2000), imitation is disrupted by even minor changes in the color or form of the puppet when they are tested with a novel puppet (Hayne et al., 1997, 2000). When tested in the same procedure, however, 18-month-olds are resilient to some changes in the context or features of the puppet, but if the perceptual dissimilarity of the puppet from encoding to retrieval is increased further, then once again memory retrieval by 18-month-olds is disrupted (Hayne et al., 1997).

Memory generalization can also be enhanced in very young infants by exposing them to different stimuli or to different contexts during the original encoding (Fagen et al., 1984; Greco et al., 1990; Amabile and Rovee-Collier, 1991; Rovee-Collier and Dufault, 1991; Learmonth et al., 2004). For example, the onset of independent locomotion (crawling) is both highly variable among infants and allows infants to explore their environment and encounter different objects and different contexts. Herbert et al. (2007) examined memory generalization in 9-month-old infants and found that infants who were not yet crawling (non-crawlers) as well as infants who were experienced crawlers (crawlers) were able to recall the target actions if the stimuli and context at test matched those presented during demonstration (cued recall). When infants were tested with different target stimuli in a different context, only crawlers were able to exhibit memory generalization.

Considering the daily bilingual language environment, bilingual infants are exposed to more varied speech patterns than monolingual infants and are also presented with more opportunities to encode information in a variety of language contexts. This may contribute to the demonstrated enhancement of memory generalization (Brito and Barr, 2012, 2014; Brito et al., 2014), as bilingual infants may have more practice making more associations and taking advantage of a wider range of retrieval cues.

### Working memory

Working memory refers to the ability to hold information in mind and update this information while executing a task (Morris and Jones, 1990; Smith and Jonides, 1998). The “updating” component of working memory is considered to be crucial as “this updating function goes beyond the simple maintenance of task-relevant information in its requirement to dynamically manipulate the contents of working memory” (Miyake et al., 2000, p. 57), distinguishing working memory from short-term memory which passively stores information. Working memory is critical for both cognitive development and academic achievement, and working memory abilities have been correlated with language and mathematical abilities (Gathercole et al., 2004; Passolunghi et al., 2007; Swanson and Kim, 2007).

Infant working memory is typically measured using looking A-not-B or delayed response tasks that focus on infants' abilities to remember the spatial location of hidden objects (Diamond, 1990). During these tasks, infants constantly form and update temporary representations of objects and their locations (Reznick, 2007). Unlike adult working memory tasks, infant working memory tasks must be non-verbal and often rely on additional cognitive skills such as inhibition and attention (see Diamond, 1990). During working memory tasks, infants must inhibit looking toward a previously rewarded hiding location and look at the current correct location, requiring a significant amount of sustained attention and inhibition throughout the task (Diamond et al., 1997; Bell and Adams, 1999). Due to reliance on other cognitive processes, previous studies have associated these infant working memory tasks with executive functioning skills and the dorsolateral prefrontal cortex (Diamond, 1990; Baird et al., 2002). More recently, slightly more complex tasks have been developed that measure both maintenance and updating functions of spatial working memory, such as the *Spin the Pots* task (Hughes and Ensor, 2005) and performance on this task has been related to the quality of parent-child interactions (Bernier et al., 2010).

There has been limited evidence of a bilingual advantage in working memory within the literature. Engel de Abreau (2011) followed 6-year-old monolingual and bilingual children longitudinally over a period of 3 years and reported no difference between groups on simple and complex working memory tasks. Morales et al. (2013) examined working memory performance in 5-year-old monolingual and bilingual children using the Simon task and a computerized variant of the Cori blocks task, which is used to measure visuospatial working memory. Although their results demonstrated a bilingual advantage in working memory, this advantage was related to other executive function demands of the task and may not be an advantage specific to working memory.

### Present study

The current study aimed to answer two questions. The first was to test the specificity of the bilingual advantage in memory. Is this advantage a global enhancement of memory processes including working memory, cued recall, and memory generalization, or one specific to memory generalization? Second, how does performance in each task compare across toddlers exposed to different numbers of languages?

In a previous study of 18-month-olds (Brito et al., 2014), although the linguistic environment for the trilingual group was thought to be more variable than the bilingual group, the trilingual infants did not demonstrate memory generalization across the perceptually different stimuli and performed similarly to the monolingual group of infants. The *threshold level hypothesis* (Cummins, 1976, 1979) states that a certain level of linguistic understanding or ability is necessary for the cognitive advantages of bilingualism to present itself, and this threshold may not have been reached by 18-months of age. Additionally, Brito et al. (2014) reported no difference between language groups on a measure of simple working memory. More complex working memory abilities like updating representations develop in the second year of life (Gathercole, 1998; Garon et al., 2008) and differences between groups may be present later in development. To answer these questions, 24-month-old monolingual, bilingual, and trilingual toddlers were tested on measures of cued recall, memory generalization, and working memory. Given that parent-child interactional quality has recently been associated with measures of executive functioning during toddlerhood (Carlson, 2009; Bernier et al., 2010), parent-child interactional quality, assessed using a picture-book reading task, was also examined. Finally a measure of productive vocabulary was given to compare language abilities across groups.

## Methods

### Participants

Our final sample included 18 toddlers in the monolingual group, 18 toddlers in the bilingual group, 14 toddlers in the trilingual group, and 14 monolingual toddlers in the baseline control group (32 male, 32 female; *M* age = 24.50 months, *SD* age = 0.39) recruited in Washington, DC. Ten additional toddlers were excluded from the analyses because of experimental error (*n* = 4) or infant fussiness (*n* = 6). Parents were primarily Caucasian (*n* = 41) or mixed race (*n* = 21), middle- to high-income, and well educated, with no differences between the monolingual, bilingual, trilingual, or baseline groups on mean parental educational attainment [*F*_(3, 59)_ = 2.1, *p* = 0.11] or mean rank of socioeconomic index [*F*_(3, 53)_ = 0.49, *p* = 0.69], see Table 1.

**Table 1:** **Means (standard deviations) for demographic variables**.

	**Child age in months**	**Parental education in years**	**Rank SEI**
Monolingual	24.43 (0.50)	17.44 (1.15)	75.28 (17.38)
Bilingual	24.56 (0.26)	17.67 (0.77)	75.62 (12.41)
Trilingual	24.46 (0.38)	18 (0.0)	76.21 (13.41)
Baseline	24.50 (0.39)	18 (0.0)	81.30 (13.11)

Bilingual children were defined as those who had been exposed to two languages on a daily basis from birth and trilingual children were defined as children who had been exposed to three languages on a daily basis from birth. A child's language exposure was measured by an adapted version of the Language Exposure Questionnaire (Bosch and Sebastián-Gallés, 2001) to obtain specific estimates of the child's exposure to each language from all possible language partners (e.g., parents, grandparents). Average first language (L1) exposure for the English monolingual group was 98% (some children were minimally exposed to a second language via a secondary caregiver). Average L1 exposure for the bilingual group was 69%; range of second language (L2) exposure for the bilingual group was between 25 and 50%. For the trilingual group, average L1 exposure was 48%, average L2 exposure was 33%, and average L3 exposure was 19%. Range of L2 exposure for the trilingual group was between 25 and 40% and range of L3 exposure was between 10 and 30%. See Table 2 for description of languages and language percent exposure for each group. All children in the baseline control group were only exposed to English. Past studies examining the influence of multilingualism on memory generalization have found bilingual advantages are not dependent on exposure to specific language pairs (Brito and Barr, 2012, 2014; Brito et al., 2014), therefore type of language exposed to was not controlled for.

**Table 2:** **Description of languages**.

	**Monolingual**	**Bilingual**	**Trilingual**
L1 languages	English (*n* = 18)	English (*n* = 13)	English (*n* = 5)
		Spanish (*n* = 4)	Spanish (*n* = 4)
		French (*n* = 1)	Hebrew (*n* = 1)
			Arabic (*n* = 1)
			Farsi (*n* = 1)
			French (*n* = 1)
			Portuguese (*n* = 1)
L1 avg. percent	98% (range = 90–100)	69% (range = 50–75)	48% (range = 35–65)
L2 languages	Spanish (*n* = 3)	Spanish (*n* = 6)	Spanish (*n* = 5)
	French (*n* = 1)	English (*n* = 5)	German (*n* = 3)
	Thai (*n* = 1)	German (*n* = 2)	Portuguese (*n* = 2)
		Italian (*n* = 2)	Turkish (*n* = 1)
		Hebrew (*n* = 1)	French (*n* = 1)
		Chinese (*n* = 1)	English (*n* = 1)
		Portuguese (*n* = 1)	German (*n* = 1)
L2 avg. percent	2% (range = 0–10)	31% (range = 25–50)	33% (range = 25–40)
L3 languages	NA	NA	English (*n* = 8)
			Spanish (*n* = 2)
			Hebrew (*n* = 1)
			Farsi (*n* = 1)
			Danish (*n* = 1)
			French (*n* = 1)
L3 avg. percent	NA	NA	19% (range = 10–30)

### Apparatus

#### Deferred imitation

The stimuli for the cued recall and generalization tasks were identical to the ones used in previous studies of deferred imitation and memory at 24-months of age (Herbert and Hayne, 2000). There were two types of stimuli (an animal and a rattle) with two versions of each type. The stimuli were constructed so that the same three target actions could be performed with each version of each stimulus, see Table 3.

**Table 3:**
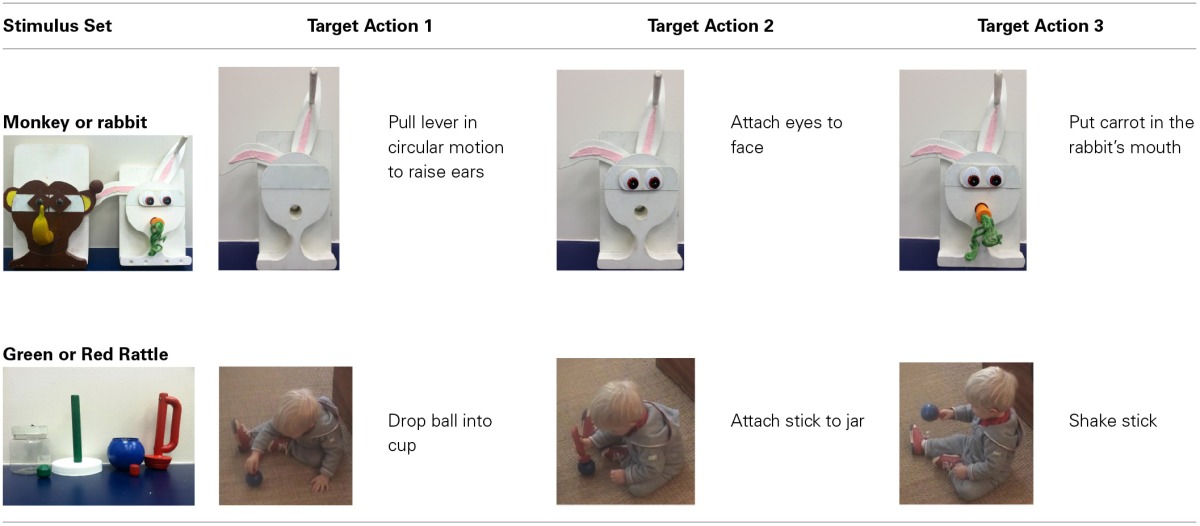
**Target actions for each stimuli set at 24-months**.

*Same target actions were completed with the alternate stimulus. For the monkey a banana was used in the 3rd step; for the green rattle the ball was pushed through into the cup (Herbert and Hayne, 2000)*.

The stimuli for the rabbit consisted of two plastic eyes (3 × 2 cm) with eyelashes attached to a 9 × 6 cm piece of plywood with Velcro on the back, a 12-cm orange wooden carrot with green string attached to the top, and a white circle of wood (the head, 15 cm in diameter) mounted horizontally on a white rectangular wooden base (30 × 20 cm). A 3-cm diameter hole was drilled at the bottom of the head, and a 5 × 15 cm piece of Velcro was attached to the top of the head. Two white “ears” (20 × 5 cm) decorated with stripes of pink felt were hidden behind the head. A 10-cm wooden stick attached to the top of the right ear allowed the ears to be pulled up from behind the head in a circular motion to a point above the head. The stimuli for the monkey consisted of two plastic eyes (2.5 cm in diameter) that were attached to a piece of brown plywood in the shape of two diamonds joined at the center (11.5 cm in width, 6.5 cm in height), with brown Velcro on the back; a 20.5-cm yellow plastic banana; and a brown wooden base (22 × 38 cm). A 4-cm hole was drilled at the bottom of the head, and a 5 × 18 cm piece of brown Velcro was attached to the top of the head. Two brown ears (3.5 × 7 cm) decorated with a piece of yellow felt were hidden behind the head. A 3-cm lever with a wooden button (3.5 cm in diameter) on the top, attached to the right ear, allowed the ears to be pulled up from behind the head in a circular motion to the side of the head.

The stimuli for the green rattle consisted of a green stick (12.5 cm long) attached to a white plastic lid (9.5 cm in diameter), with Velcro attached to the underside of the lid; a round green bead (3 cm in diameter × 2.5 cm in height); and a clear plastic square cup with Velcro around the top (5.5 cm in diameter × 8 cm in height). The opening of the plastic cup (3.5 cm in diameter) was covered with a 1 mm black rubber diaphragm, with 16 cuts radiating from the center. The stimuli for the red rattle consisted of a red D-shaped handle (gap between stick and handle = 1.5 × 8 cm) attached to a red wooden stick (12.5 cm long) with a plug on the end, which fitted into a blue plastic cup with a hole cut in the top (4 cm in diameter); and a red wooden bead.

#### Working memory

**Figure 1:**
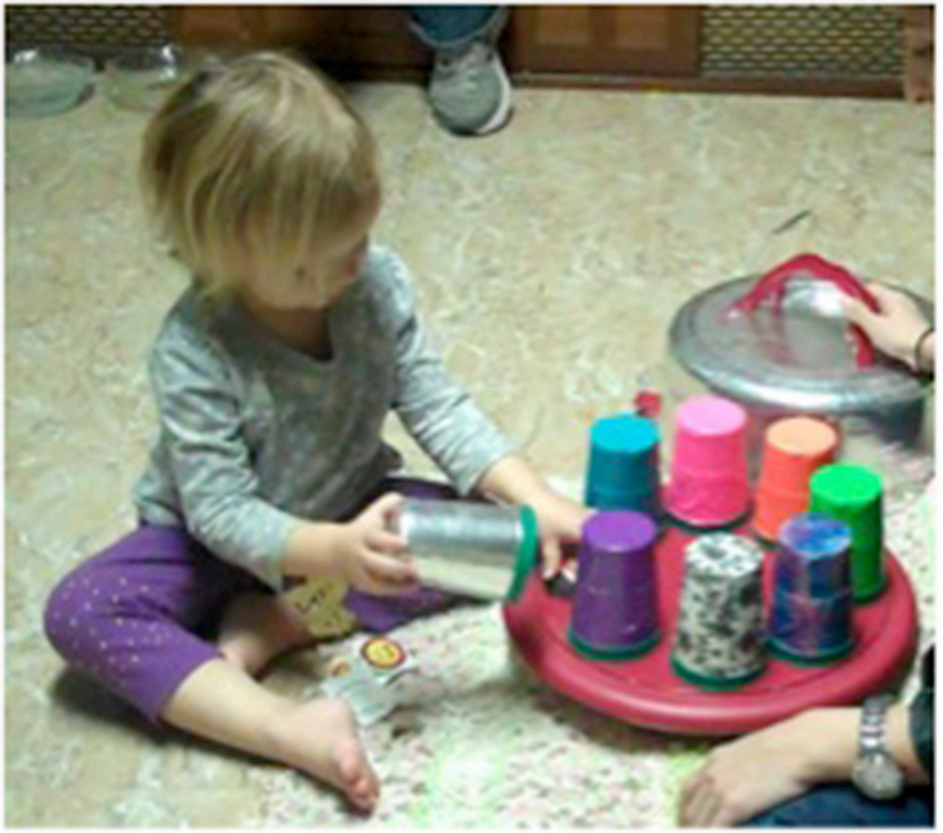
**Picture of 2-year-old completing a trial in the *Spin the Pots* WM task**.

The *Spin the Pots* (Hughes and Ensor, 2005; Bernier et al., 2010) task was used as a measure of working memory. Eight distinctly colored opaque cups, six attractive stickers, and a lazy Susan with a cover were used in this task. All eight cups fit inside the lazy Susan in a circle with equal spacing between them. An opaque cover was used to cover the cups in between trials and had a handle on top of the cover in order to easily cover and uncover the lazy Susan, see Figure 1.

#### Parent-child interaction

A joint picture-book reading task was used to assess parent-child interactional quality. The picture books “ABCs,” and “From 1 to 10” by Richard Scarry and “Good Night Gorilla” by Peggy Rathmann were selected due to the variety of colorful objects and different scenarios presented within the books. All words and phrases were covered over with opaque tape to ensure that parental vocalizations and behaviors were not constrained to the written text.

#### Self-report measures

The caregiver was asked to complete a general information questionnaire (assessing rank Socioeconomic Index, parental education, and language) as well as the MacArthur Communicative Development Inventory: Words and Sentences Short Form (MCDI) to measure children's productive vocabulary (Fenson et al., 2000). Due to the wide variety of languages, language specific vocabulary measures were not feasible. For the bilingual and trilingual children, the caregiver was asked to fill out the same form for all languages, marking the words the child could produce and in which language (e.g., for a Spanish-English bilingual child: English, Spanish, or both).

### Procedure

All protocols were approved by the Georgetown University IRB. All stimuli and deferred imitation procedures were identical to Herbert and Hayne (2000, Exp. 1A). The children were seen on two consecutive days, with the demonstration of target actions for the deferred imitation tasks occurring on the first day and children's ability to recall target actions tested on the following day (24-h delay ± 4 h). The parent-child interaction task and surveys (general information questionnaire and vocabulary measure) were completed on the first day and the working memory task was given on the second day.

On the first day, the parent-child interaction task was completed first. Parents were given all three picture books and were instructed to “read to your child as you normally would at home.” During this unstructured task, parents and children could select any of the books at any time and book order was not specified. After the 5-min book reading task, the demonstration portion of the deferred imitation task began. During the demonstration of the target actions, children sat on the floor with the caregiver, across from the experimenter. The experimenter performed the three target actions with one version of each stimulus type, and the entire demonstration lasted approximately 60 s. The experimenter did not describe the stimuli or the target actions, and the child was not allowed to touch the stimuli. The order of presentation of the stimulus sets was counterbalanced across participants. After the demonstration, the caregiver was asked to complete the general information questionnaire and the vocabulary measure.

On the second day, children were first tested on the deferred imitation task. Children were tested with one set of stimuli that had been used in the original demonstration (cued *recall*) and one set of stimuli that was perceptually different from the one seen during demonstration (*generalization*) but that required the same target actions. The two types of stimuli (rattle or animal) and the order of presentation at test (cued recall or generalization) were counterbalanced across children. During the test, children were given the first set of stimuli and the experimenter encouraged the child to interact with the stimuli for 60 s from the time the child first touched the stimuli. Children were then given the second set of stimuli and then given another 60 s to interact with that stimulus. The test procedure was identical for the experimental and baseline control groups; however, children in the baseline control group were not shown the demonstration of the target actions on the first day. Rather, the baseline group was only seen for one session and simply shown each stimulus type, one at a time, at test to assess the spontaneous production of the target actions.

Next, the working memory task was completed. For the *Spin the Pots* task, the experimenter encouraged the child to place the six attractive stickers under six of the eight brightly colored cups, leaving two cups empty. After all stickers were hidden, the experimenter showed the child the two cups that did not have a sticker and said, “Look, no stickers under these cups!” The opaque cover was placed over all the cups on the lazy Susan and the entire tray was spun 180 degrees. The experimenter uncovered the cups and instructed the child to find one of the stickers. If the child found a sticker, the experimenter praised the child, the sticker was set aside or given to the child's caregiver, and the lid was replaced and the tray was spun 180° again. After each trial, the tray was spun 180° to counterbalance the position of the cups. If the child did not find a sticker, the experimenter gave appropriate feedback (e.g., “no sticker there, let's try again”) and the lid was replaced and the tray was spun 180° again. The child had up to 16 trials to find all six stickers. This task required the child to hold the location of the cups that *did not* have stickers in mind and to update this memory after each trial. The task ended when the child found all six stickers or reached 16 trials.

### Coding

#### Deferred imitation

For both cued recall and generalization, one coder scored each videotaped test session for the presence of the three target actions during the 60 s test period for each stimulus type. The number of individual target actions produced during the 60 s after the child first touched the stimuli was summed to calculate the imitation score (range = 0–3) for each stimuli type. Each child had an imitation score for stimuli that was identical to the demonstration session (cued recall) or perceptually different from the demonstration session (generalization). A second independent coder scored 40% of the videos to determine reliability of the ratings; there was an inter-rater reliability kappa of 0.88.

#### Working memory

For the *Spin the Pots* task, each child was given a working memory score, a trial rate score, a perseveration score, and a correction score. The working memory score was calculated as 16 minus the number of errors made if the child found all six stickers or completed all 16 trials, with larger scores indicating better working memory. If the child did not find all six stickers or complete all 16 trials, their score was calculated based on the number of stickers found. This was to ensure that a child's score would not be inflated due to inability to complete the task. For example, a child who finds all six stickers without making any errors would obtain a perfect score of 16. Another child who finds all six stickers but makes five errors (by choosing an empty cup) would obtain a score of 11. Finally, a child who completes all 16 trials but only finds three stickers would obtain a score of three. The number of times the child chose a cup that was selected on the previous trial (perseveration) and the number of times the child started to choose an incorrect cup but then switched to the correct cup (correction) were also calculated. A second independent coder scored 40% of the videos to determine reliability of the ratings; there was an inter-rater reliability kappa of 0.99.

#### Parent-child interaction

For the parent-child interaction task, one coder scored each videotaped dyadic interaction on three subscales of Emotional Responsiveness (ER): Shared Focus, Parental Warmth, and Turn-Taking. These measures were derived from past studies on parent-child interactions during joint book-reading sessions (Bornstein, 1985; DeLoache and DeMendoza, 1987; Bornstein and Tamis-LeMonda, 1989; Senechal et al., 1995; Bus et al., 2000). Each 5-min video was rated on a 0–4 scale, with 0 being low and 4 being high and ratings occurring at ½ point intervals. A rating was made every minute and then averaged across the 5-min session, resulting in a score for each subscale. Shared Focus (SF) describes the sense of togetherness and joint focus on the book reading task between parent and child; Parental Warmth (PW) is the degree of sensitivity that the parent displays toward his or her child's affective cues, such as appropriateness of reactions, positive affect, and tone of voice; and Turn Taking (TT) is the amount of verbal and non-verbal back-and-forth interaction between the parent and child. Thirty percent of the videos were double-coded for ER and the overall intra-class reliability was 89%.

## Results

A preliminary analysis examining associations between parental education, family rank SEI, child gender, stimuli type, or stimuli order and imitation performance yielded no main effects or interactions for any of the three outcomes of interest (cued recall, generalization, or working memory); therefore the data were collapsed across these variables in the following analyses. For children in the deferred imitation baseline control group, a within-subjects *t*-test indicated no differences in performance by stimuli type (animal vs. rattle); therefore these scores were averaged to create the baseline score.

**Figure 2:**
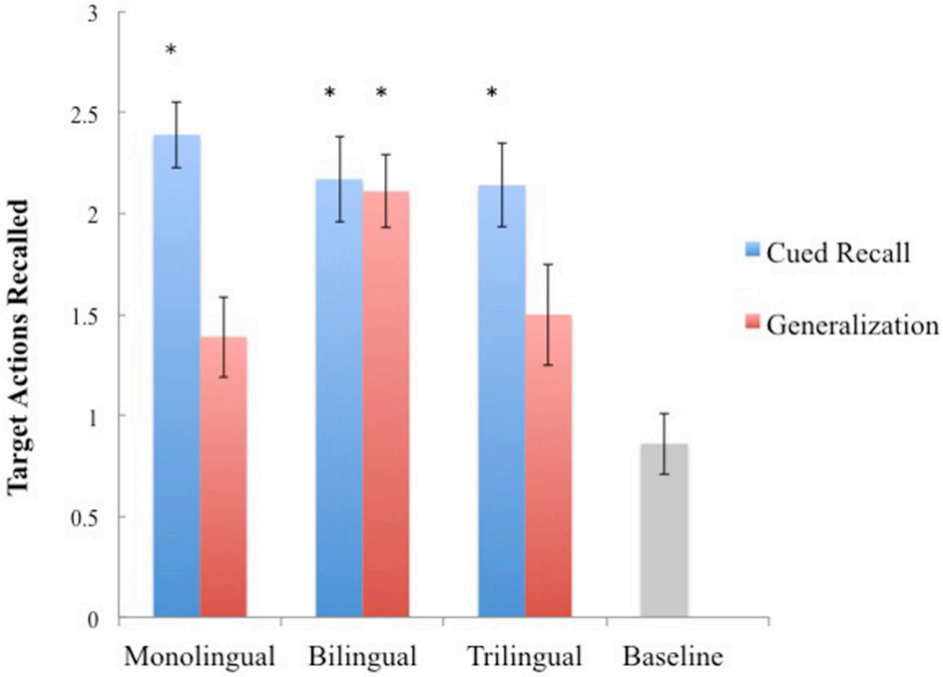
**Mean imitation scores across language groups with error bars indicating standard error of the mean**. An asterisk indicates that performance significantly exceeds that of the baseline control group.

The three outcomes of interest were initially analyzed separately to examine differences between language groups. Cued recall scores were examined first, and a One-Way ANOVA yielded significant differences between all four groups, *F*_(3, 60)_ = 14.03, *p* < 0.001, ηρ^2^ = 0.41. Deferred imitation is operationally defined as performance by the experimental group that significantly exceeds performance by the baseline control group. A *post-hoc* Student Newman-Keuls (SNK, *p* < 0.05) analyses across all four groups indicated that the monolingual (*M* = 2.39, *SD* = 0.70), bilingual (*M* = 2.17, *SD* = 0.79), and trilingual (*M* = 2.14, *SD* = 0.77) groups all significantly exceeded the performance of the baseline control group (*M* = 0.86, *SD* = 0.57), suggesting that all three groups were able to recall the target actions after a 24-h delay when the stimuli were identical from encoding to retrieval, see Figure 2. Examining only the experimental groups, a One-Way ANOVA indicated no significant differences between language groups for cued recall scores, *p* = 0.58.

Next, memory generalization scores were examined and again a One-Way ANOVA yielded significant differences between all four groups, *F*_(3, 60)_ = 6.74, *p* = 0.001, ηρ^2^ = 0.25. This time the SNK *post-hoc* analyses indicated that only the bilingual group (*M* = 2.11, *SD* = 0.76) significantly outperformed the baseline control group (*M* = 0.86, *SD* = 0.57). There were no significant differences between the baseline control group and the monolingual group (*M* = 1.39, *SD* = 0.85), or the trilingual group (*M* = 1.50, *SD* = 0.94). Examining only the experimental groups, a One-Way ANOVA indicated a significant difference between language groups for memory generalization scores, *F*_(2, 60)_ = 3.73, *p* = 0.031, ηρ^2^ = 0.14. Unlike SNK *post-hoc* analyses, Scheffe *post-hoc* tests allow for all possible simple and complex comparisons; therefore Scheffe *post-hoc* analyses were utilized to compare the performance of monolingual and trilingual groups to the bilingual group performance. Analyses indicated a significant difference between the monolingual and bilingual groups, *p* = 0.04, but no difference between the bilingual and trilingual groups, *p* = 0.14. These results indicate that, when compared to the baseline control group, only the bilingual group was able to successfully recall the target actions when the perceptual features of the stimuli changed from encoding to retrieval, but bilingual scores were not statistically different from trilingual scores when only comparing across experimental groups, see Figure 2.

Finally, we examined working memory performance by language group. A One-Way ANOVA yielded no significant differences between language groups on *Spin the Pots* scores, *p* = 0.85, perseveration frequency, *p* = 0.17, or correction frequency, *p* = 0.90, but performance on this working memory task was highly variable, see Table 4.

**Table 4:** **Means (standard deviations) for *Spin the Pots* working memory task**.

	***Spin the Pots* score**	**Perseveration score**	**Correction score**
Monolingual	6.75 (3.53)	0.31 (0.60)	0.13 (0.34)
	Range = 2–13	Range = 0–2	Range = 0–1
Bilingual	6.76 (3.03)	0.82 (0.88)	0.18 (0.39)
	Range = 3–12	Range = 0–3	Range = 0–1
Trilingual	7.36 (2.42)	0.73 (0.91)	0.18 (0.41)
	Range = 5–13	Range = 0–2	Range = 0–1

We also examined differences in productive vocabulary scores and parent-child emotional responsiveness scores by language groups. As recommended by studies measuring vocabulary scores using the MCDI with bilingual populations (Hoff et al., 2012), the raw MCDI scores were analyzed instead of the percentile scores. Controlling for gender, there was a significant difference between groups on English vocabulary scores, *F*_(2, 43)_ = 9.60, *p* < 0.001, with a *post-hoc* tests indicating a significant difference between monolingual and both bilingual English scores (*p* = 0.005) and trilingual English scores (*p* = 0.001), but no difference between bilingual and trilingual English scores (*p* = 0.60). Only a trend was obtained between language groups on MCDI scores when raw scores for all languages were combined (*p* = 0.07), with *post-hoc* tests indicating no significant differences between monolingual and bilingual scores (*p* = 0.51) or bilingual and trilingual scores (*p* = 0.36), but a trend when comparing monolingual and trilingual scores (*p* = 0.06). Although the use of one vocabulary inventory standardizes the measurement of productive vocabulary across languages, it is worth noting that language specific inventories vary by the acquisition of common words in that specific language and only using the English form may underestimate the productive language skills of the multilingual children. For Emotional Responsiveness, 5 monolingual, 6 bilingual, and 7 trilingual videos were unable to be coded (due to the task not being administered, dyads not completing the task or camera malfunction), but there was no difference in cued recall, generalization, or working memory scores for children who completed vs. did not complete the book reading task, *p*'s > 0.11. We found no difference between language groups on overall emotional responsiveness, *p* = 0.39, or any of the individual subscales, *p*'s > 0.44, see Tables 5, 6.

**Table 5:** **Means (standard deviations) for MCDI vocabulary raw scores**.

	**English**	**All languages**
Monolingual	66.44 (14.55)	NA
Bilingual	40.67 (26.88)	59.28 (15.98)
Trilingual	31.90 (20.45)	49.20 (24.24)

**Table 6:** **Means (standard deviations) for emotional responsiveness book-reading task**.

	**Parental Warmth**	**Turn-taking**	**Shared Focus**	**Total ER**
Monolingual	2.36 (0.37)	2.32 (1.12)	2.55 (0.78)	7.23 (1.97)
Bilingual	2.68 (0.37)	2.61 (0.41)	2.68 (0.37)	7.57 (0.66)
Trilingual	2.21 (0.93)	2.38 (0.85)	2.21 (0.93)	6.50 (2.05)

Examining correlations between the memory tasks and parent-child interaction scores (total ER) yielded no significant correlations across tasks. As shown in Table 7, none of the memory tasks (cued recall, generalization, working memory) correlated with one another, and they also did not correlate with parent-child interaction (Total ER) scores. Consistent with studies at 18-months (Brito and Barr, 2012), memory generalization was associated with percent exposure to the second language (%L2). A perfectly balanced bilingual would have a %L2 of 50%, a perfectly balanced trilingual would have a %L2 of 33%, and a monolingual with no exposure to a second language would have a %L2 of 0%. Here we find that only memory generalization is associated with %L2, where higher second language exposure is correlated with higher memory generalization scores.

**Table 7:** **Correlations between tasks**.

	**%L2**	**Cued recall**	**Memory generalization**	**Working memory**	**Total ER**
%L2	–				
Cued recall	−0.15	–			
Memory generalization	0.33^*^	−0.005	–		
Working memory	0.002	0.14	0.05	–	
Total ER	0.22	0.10	0.13	0.28	–

Note:

* p < 0.05, ^**^p < 0.01, ^***^p < 0.001.

## Discussion

Overall, these results replicate past studies (Herbert and Hayne, 2000; Brito and Barr, 2012, 2014; Brito et al., 2014) and support the hypothesis that experience with two languages from birth enhances memory generalization performance, with higher second language exposure associated with higher memory generalization performance. This study also extends prior research to demonstrate that it is not the inability to recall information on the part of the monolinguals and trilinguals that differentiates them from the bilingual group. Each toddler was tested with one stimulus that was identical from encoding to retrieval and one stimulus that was different. Groups did not differ in the cued recall condition when tested with the same stimuli as had been presented during the demonstration. The bilingual children performed at an equal level to the monolingual and trilingual groups. Although both the monolingual and trilingual groups were able to recall the target actions when tested with identical stimuli, memory retrieval performance decreased for these groups when the perceptual features of the stimuli changed from demonstration to test. It is important to note that while the trilingual group did not outperform the baseline control group, the trilingual group performance did not significantly differ from either the monolingual or bilingual groups. Like the cued recall condition, there were no significant group differences in working memory performance either, suggesting a very specific bilingual advantage for memory generalization during infancy. Finally, the current study included a measure of parent-child interaction, to test the possibility that overall enhanced memory skills were associated with higher quality parent-child interactions, but no differences were found across language groups and parent-child interaction was not associated with memory performance.

Researchers have argued for a parallel association between initial perceptual processing of information and memory organization (Bhatt and Rovee-Collier, 1996, 1997). A dissociation has been found where cognitive load can influence relational information in memory but does not affect the encoding of featural information (Bhatt and Rovee-Collier, 1997). Relational memory, in comparison to memory for object features, may indeed be cognitively challenging for younger children. Past research in perceptual development has demonstrated that children shift from attention to parts of objects to more configural or whole representations with both increasing age and expertise with objects (Davidoff and Roberson, 2002; Pereira and Smith, 2009). This perceptual shift may develop in parallel with a cognitive shift toward more attention and understanding of relational structures (Kotovsky and Gentner, 1996; Augustine et al., 2011). Furthermore, this development of relational reasoning may be influenced by differences in cultural practices. Kuwabara and Smith (2012) tested the hypothesis that children growing up in Eastern cultures, relative to those growing up in Western cultures, are more advanced in relational matching tasks as opposed to object search tasks. Results indicated an advantage in relational matching for 4-year-old children growing up in Japan, with age-matched peers from the U.S. outperforming the Japanese children at visual search tasks. These results demonstrate how early environmental variations can shape the developmental trajectory of different cognitive domains.

The current study demonstrates an advantage for bilingual toddlers in memory generalization, but not other memory processes, and this shifted cognitive trajectory may be the result of two mechanisms. First, because bilingual toddlers are exposed to a more varied speech input, as a result of statistical learning, bilingual toddlers may be more attuned to detecting and recalling patterns in both auditory and perceptual stimuli. This has been demonstrated within the bilingual literature (Weikum et al., 2007; Sebastián-Gallés et al., 2012; Werker, 2012) and past studies have shown that exposure to different stimuli or contexts enhance memory generalization in very young infants (Fagen et al., 1984; Greco et al., 1990; Amabile and Rovee-Collier, 1991; Rovee-Collier and Dufault, 1991; Learmonth et al., 2004). The additional daily exposure to different languages may influence a child's ability to make relational associations between stimuli and form hierarchical memories earlier in development, leading to enhanced memory generalization.

Additionally, Diamond et al. (1994) have suggested that the prefrontal cortex is involved in the processing of relational information, but not in the processing of individual features (Diamond et al., 1994). Bilingual advantages have been found at 7-months of age for processes that require earlier development of the prefrontal cortex (Kovács and Mehler, 2009) and the daily monitoring of multiple languages may require additional recruitment of the executive function areas of the brain in order to successfully acquire two or more languages. In this case, the bilingual advantage in memory generalization may be due to enhancement of the prefrontal cortex and, subsequently, the ability to process relational information earlier in development.

Examining the results from the trilingual group, these hypotheses (advantages in memory generalization due to increased variation in language input and daily monitoring of multiple languages) were not supported. Although trilingual toddlers were unable to defer imitation in the generalization condition, they were able to perform as well as the monolingual and bilingual toddlers in the cued recall condition. Exposure to three languages does not seem to be a disadvantage for encoding featural information, but perhaps the cognitive load of processing more than two languages influences relational information in memory. Trilingual children, in theory, should be exposed to a more linguistically diverse environment leading to heightened awareness of multiple languages. Like Brito et al. (2014), all trilingual toddlers in the current study were learning three languages from birth and the majority of the trilinguals heard two minority (or non-community) languages in the home from their parents and were exposed to the majority or community language outside of the home or from overheard speech between the parents. Our results from the trilingual group contradict our hypotheses, but it is possible that the low and uneven exposure to the third language impeded the young child's ability to detect patterns within their languages enough to enhance memory generalization abilities. Consistent with the threshold level hypothesis (Cummins, 1976, 1979), trilinguals may need extended cumulative exposure to their different languages in order to capitalize on this cognitive advantage. Examining differences in memory generalization performance between more balanced trilinguals (e.g., 33% exposure to each language) vs. unbalanced trilinguals (e.g., 45% L1, 45% L2, 10% L3) who have a more similar language exposure profile to bilinguals may clarify this mechanism. Within the current study, when dividing the trilingual group into higher or lower L2 percent exposure, a trend is found for a difference in memory generalization performance (*p* = 0.08) with unbalanced bilinguals having higher memory scores, but the small sample size of our trilingual group does not permit further exploration of this hypothesis. More research with larger sample sizes is necessary to understand how language exposure influences both language acquisition and cognitive development. Furthermore, understanding how code switching or mixing of languages contributes to these bilingual cognitive advantages will provide additional insight into the interaction between multiple language exposure and early cognitive development.

Although consistent with past research (Engel de Abreau, 2011), the limited evidence of a bilingual advantage in working memory in the current study may be due to a limitation in the task. The *Spin the Pots* task produced a range of scores but the mean for each group was less than half of the possible maximum score of 16, indicating that these toddlers had some difficulty with this task. Although we were looking for a more complex task to observe differences between language groups, this working memory task may have been too difficult for the toddlers to complete. Past studies (Hughes and Ensor, 2005; Bernier et al., 2010) have used the *Spin the Pots* working memory task within a battery of measures, and not as a stand-alone measure of working memory, and this may have restricted the variability of scores needed to produce differences between groups. Limitations in sample size may have also masked potential differences in memory performance between language groups. We are currently testing larger samples of children to examine differences between children with varying working memory capacities in relation to other memory or executive function tasks. Although the task is not without limitations, this task does help to measure basic abilities to hold information in mind, and was crucial to provide further evidence that these differences between language groups were attributed to the ability to generalize across perceptual cues and not short-term or working memory capacity. Additionally, future studies should examine correlations between memory tasks and more unstructured measures of parent-child interactions. The structured nature of the book-reading task in the current study may have led to uniformly moderate emotional responsiveness scores across all language groups. Past studies have reported that routines occur when parents read new books to their children (Senechal et al., 1995), and the reduced variability in non-verbal behaviors by both the children and parents may have contributed to the lack of group differences. While the aim of the current study was in examining non-verbal interactions, future studies should examine the amount of language switching demonstrated by parents of bilingual and trilingual children to assess the degree to which switching between languages in the home influences bilingual cognitive advantages.

This study adds to the scant literature examining links between multilingualism and cognitive development during infancy (Kovács and Mehler, 2009; Poulin-Dubois et al., 2011; Sebastián-Gallés et al., 2012) and together, these findings make an important contribution to understanding the interactions between cognitive domains early in development. Spear (1984) proposed that what infants of all species learn and remember at any time in development is determined by the ecological challenges posed by their current environment and the survival value of responding successfully to them. When considering the basis for a bilingual cognitive advantage, future studies must take into account the bicultural environment in which children are raised. Being able to read and write in more than one language opens up new literatures, traditions, and ideas to bilingual children and often fosters greater openness to other cultural groups (Cummins, 1989). Bilingual children are not only switching between languages, but are also switching between and generalizing across cultural contexts, such as different home and school environments, rules, customs, values, and expectations (Javier, 2007; Kuwabara and Smith, 2012). Differences in child-rearing culture or customs may contribute to the development of cognitive control and memory generalization. Languages that are more disparate to one another, either linguistically or culturally (e.g., English and Japanese), may influence bilingual advantages in memory generalization and other non-linguistic cognitive tasks, but the association between linguistic environment and memory flexibility within the parameters of this study appear to be robust and dependent on exposure to two languages. By studying the development of multilingual children, particularly early in development, we stand to expand our understanding of the role of language and culture in cognitive development.

### Conflict of interest statement

The authors declare that the research was conducted in the absence of any commercial or financial relationships that could be construed as a potential conflict of interest.
